# iPSC‐Derived MSC Secretome as a Protective and Restorative Modality for Atopic Dermatitis

**DOI:** 10.1155/sci/4412511

**Published:** 2025-12-11

**Authors:** Seungah Yoo, Hyun Jin Baek, Narae Park, Yoojun Nam, Yeri Alice Rim, Ji Hyeon Ju

**Affiliations:** ^1^ Department of Dermatology, Seoul St. Mary’s Hospital, College of Medicine, The Catholic University of Korea, Seoul, Republic of Korea, catholic.ac.kr; ^2^ YiPSCELL Inc., L2 Omnibus Park, Banpo-daero, Seocho-gu, 222, Seoul, Republic of Korea; ^3^ Department of Biohealth Regulatory Science, Sungkyunkwan University, Suwon, Republic of Korea, skku.edu; ^4^ CiSTEM Laboratory, Catholic iPSC Research Center, Seoul St. Mary’s Hospital, College of Medicine, The Catholic University of Korea, Seoul, 06591, Republic of Korea, catholic.ac.kr; ^5^ Division of Rheumatology, Department of Internal Medicine, Seoul St. Mary’s Hospital, Institute of Medical Science, College of Medicine, The Catholic University of Korea, Seoul, Republic of Korea, catholic.ac.kr

**Keywords:** atopic dermatitis, iPSC-derived mesenchymal stem cell, regeneration, secretome, stem cell

## Abstract

**Background:**

Atopic dermatitis (AD) is a chronic inflammatory skin disease that significantly impacts quality of life. Reducing inflammation and restoring the skin barrier are key to its management.

**Objective:**

This study aimed to investigate the protective and therapeutic effects of secretory substances from induced pluripotent stem cell (iPSC)‐derived mesenchymal stem cells (iMSCs) in AD.

**Methods:**

The protective effects of iMSC secretome pretreatment were evaluated in HaCaT cells by assessing cell viability, AD biomarker expression, and cell migration. Therapeutic efficacy was examined in a 1‐chloro‐2,4‐dinitrobenzene (DNCB)‐induced AD mouse model through clinical, histological, and immunological assessments. Proteomic analyses were performed to relevant biological processes.

**Results:**

iMSC secretome significantly reduced AD‐induced cell death and AD biomarker expressions in vitro (*p*  < 0.05), with 200 μg/mL iMSC secretome promoting cell migration. In vivo, high dose (20 mg/mL) iMSC secretome alleviate clinical indicators compared to the vehicle group (*p*  < 0.05). Serum immunoglobulin (Ig) E, interleukin (IL)‐4, IL‐31, and IL‐6 levels, along with the expression of AD biomarkers in skin, were significantly decreased (*p*  < 0.05). Proteomic analyses revealed upregulation of genes involved in the regulation of immune responses and the restoration of skin barrier.

**Conclusion:**

iMSC secretome demonstrates significant anti‐inflammatory and regenerative effects, making it a promising therapeutic option for AD.

## 1. Introduction

Atopic dermatitis (AD) is a chronic inflammatory skin disorder characterized by epidermal barrier disruption and immune dysregulation. It results in persistent inflammation, severe itching, and significant disease‐related burdens for patients [1]. AD affects up to 30% of children and approximately 10% of adults worldwide [2, 3]. Its pathogenesis involves complex interactions between innate and adaptive immunity, influenced by genetic and environmental factors [4].

Managing AD remains challenging due to its chronic, recurrent nature, and fluctuating course. Current topical treatments, such as topical corticosteroids (TCSs) and topical calcineurin inhibitors (TCI), provide symptomatic relief but are associated with significant drawbacks, including systemic absorption risks with TCS and localized discomfort with TCI [5, 6]. These limitations underscore the need for novel, safe, and effective long‐term therapies.

Stem cell therapy is one of the emerging treatment modalities for AD, with mesenchymal stem cells (MSCs) showing potential due to their immunomodulatory, anti‐inflammatory, and regenerative properties [7–9]. MSC have demonstrated therapeutic benefits in autoimmune diseases such as rheumatoid arthritis and systemic lupus erythematosus and antiallergic effects in conditions such as allergic rhinitis and asthma, often associated with AD [10–13]. In AD, a phase I/IIa clinical trial of human umbilical cord blood‐derived MSC demonstrated a 55% Eczema Area Severity Index (EASI)‐50 response in moderate‐to‐severe AD patients treated with a high dose (5.0 × 10^7^) [9]. However, challenges such as donor variability, genetic instability, and risks of tumorigenicity limit their clinical applicability [14, 15].

An alternative involves the bioactive molecules that stem cells release during the culture process, which mediate paracrine effects, including cytokines, immunomodulatory factors, chemokines, growth factors, nucleic acids, lipids, and extracellular vesicles [16]. Collectively referred to as the “secretome,” this cell‐free composition represents a potent regulatory system with significant therapeutic potential. Secretome was initially expected to enhance the microenvironment and amplify the efficacy of cell‐based therapies [17]. However, owing to their complex composition—rich in extracellular vesicles containing proteins, cytokines, chemokines, enzymes, lipids, RNAs, and miRNAs—they are now being actively investigated as standalone tools for both diagnosis and treatment across a wide range of diseases [18, 19].

Secretome offers compelling advantages over direct cell transplantation. It eliminates risks of tumorigenicity and immune rejection, thus, enabling safer therapeutic applications [20]. Additionally, the secretome can be preserved for extended periods through freezing or freeze‐drying without compromising its biological activity, ensuring long‐term usability [21]. Unlike cell‐based therapies, secretome production can be efficiently scaled up in controlled laboratory environments, independent of specific tissues or donor‐derived MSC, making it a more accessible therapeutic option.

This study investigated the efficacy of the secretome from induced pluripotent stem cell (iPSC)‐derived MSC (iMSC) in AD. Our focus was on the secretome’s potential to alleviate skin inflammation, restore the epidermal barrier, and prevent disease exacerbation, aiming to provide a clinically translatable solution for managing AD.

## 2. Materials and Methods

### 2.1. iMSC Generation

1 × 10^6^ iPSCs were seeded in a 75 cm^2^ dish with E8 medium. On day 5, when the cell density reached 80%, the iPSCs were transferred to AggreWell plates at a density of 5.4 × 10^6^ cells per well to form embryoid bodies (EBs). After 1 day, the EBs were cultured in a petri dish for 4 days in E8 medium, followed by an additional 4 days in E7 medium.

Subsequently, 500 EBs were seeded into a 100 mm dish containing culture medium (Dulbecco’s modified Eagle’s medium [DMEM] low glucose, fetal bovine serum [FBS], and human fibroblast growth factor [hFGF]), supplemented with i‐Matrix and the ROCK inhibitor Y‐27632 for 7 days (passage 0). Once the cell density exceeded 80%, the cells were transferred to a fibronectin‐coated 75 cm^2^ dish.

### 2.2. Preparation of iMSC and MSC Secretome

Both iMSC and normal human bone marrow‐derived mesenchymal stem cell (BM‐MSC; Lonza) were cultured until Passage 4. At Passage 4, iMSCs were plated at 25,000 cells/cm^2^ in DMEM with 10% FBS and 0.004% FGF and cultured at 37°C for 7 days, achieving 90% confluency.

The cells were then washed five times with phosphate‐buffered saline (PBS) and cultured in phenol red‐free DMEM supplemented with 1% L‐glutamine (200 mM) and 1% sodium pyruvate (100 mM) (Gibco) for 48 h. Then the secretome was collected and freeze‐dried.

### 2.3. Preparation of iMSC Secretome for In Vitro and In Vivo Use

The lyophilized secretome was reconstituted in a solvent mixture of saline, ethanol, and glycerol (4 : 1.5 : 0.5, v/v/v) to a final concentration of 100 mg/mL. The reconstituted solution was then diluted to the appropriate concentrations for each in vitro and in vivo experiment.

### 2.4. Cell Culture and Treatment

HaCaT cells, a spontaneously immortalized human keratinocyte cell line derived from adult skin, were cultured in DMEM with 10% FBS and 1% penicillin/streptomycin solution at 37°C in a humidified atmosphere containing 5% CO_2_. AD‐like conditions were induced with interleukin (IL)‐4 (30 ng/mL), tumor necrosis factor‐alpha (TNF‐α; 10 ng/mL), and interferon‐gamma (IFN‐γ; 10 ng/mL) for 24 h.

### 2.5. Cell Viability Assay

The cytotoxicity of iMSC‐secreted substances was assessed using the Cell Counting Kit‐8 (CCK‐8) assay. HaCaT cells were seeded at a density of 3 × 10^5^ cells/well in a 96‐well plate and cultured overnight. The cells were then treated with sample concentrations ranging from 50 to 200 μg/mL for 24 h. Subsequently, 100 μg/mL of CCK‐8 solution was added, and the cells were incubated for 2 h. Optical density (OD) at 540 nm was measured using a microplate reader (BioTek).

### 2.6. Protective Effect of iMSC Secretome on Keratinocytes in AD Development

HaCaT cells were pretreated with iMSC secretome, MSC secretome, or Tacrolimus for 2 h before exposure to IL‐4, IL‐13, and IFN‐γ. Expression levels of AD biomarkers, including TNF‐α, IFN‐γ, IL‐4, IL‐13, IL‐17, IL‐22, IL‐31, chemokine (C─C) ligand 17 (CCL17), CCL22, thymic stromal lymphopoietin (TSLP), and filaggrin, were measured 24 h after challenge.

### 2.7. Wound Healing Assay

Using the Radius Cell Migration Assay kit (Cell Biolabs, #CBA‐125), HaCaT cells were preincubated with iMSC secretome for 2 h, followed by treatment with IFN‐γ (10 ng/mL), TNF‐α (10 ng/mL), and IL‐4 (30 ng/mL) for 24 h at 37°C. Migration efficacy was assessed by capturing images at 3, 6, and 24 h. The total uncovered area was quantified using ImageJ software. All experiments were conducted in triplicate.

### 2.8. Animals

Specific pathogen‐free NC/Nga male mice (4 weeks old) were obtained from Central Laboratory Animals Inc. (Seoul, Korea) and acclimatized for 1 week before the experiments. The animals were housed in a temperature‐controlled room maintained at 23 ± 3°C with 40%–60% relative humidity and a 12‐h light/dark cycle. Water and commercial rodent chow were provided ad libitum.

### 2.9. Induction of AD and Drug Treatment

Thirty mice were randomly assigned to five groups: control, vehicle, Tacrolimus (0.03% Tacrolimus ointment, Protopic, LEO Pharma, Ballerup, Denmark; 0.1 mg/mL/mouse), and secretome (5 and 20 mg/mL). For sensitization, 1% 1‐chloro‐2,4‐dinitrobenzene (DNCB, Sigma Aldrich, Saint Louis, USA) was applied to the shaved abdomen of all mice except the control group on two separate days. 2 days prior to the challenge period, dorsal hair was removed using clippers and depilatory cream. During the challenge period, 0.4% DNCB was applied to the dorsal skin and both ears three times per week to induce AD‐like lesions.

The iMSC secretome and Tacrolimus treatments were administered topically three times per week during the 18‐day challenge period. Each application consisted of 90 μL of DNCB, secretome, or Tacrolimus per mouse. 4 h after the final treatment, mice were anesthetized using an inhalation anesthesia machine (LMS Korea), and blood and tissue samples were collected for analysis.

### 2.10. Measurement of Ear Thickness and AD Severity

Ear thickness was measured twice weekly using a digital caliper (Kroeplin, Schlüchtern, Germany). The severity of dermatitis was assessed biweekly using a scoring system (0–3) for erythema, dry skin, edema/hematoma, erosion, and lichenification, with a maximum total score of 15.

### 2.11. The Enzyme‐Linked Immunosorbent Assay (ELISA)

Supernatants of blood samples collected from the eyeballs of experimental mice were used to quantify protein levels of immunoglobulin (Ig) E, IL‐4, IL‐13, IL‐31, IL‐6, and IFN‐γ. ELISA kits (R&D systems) were used according to the manufacturer’s instructions.

### 2.12. Histological and Immunohistochemical (IHC) Assessment

Dorsal skin samples were fixed in 10% neutral‐buffered formalin for histological and IHC analysis. Paraffin‐embedded tissues were sectioned into 4‐μm slices and stained with hematoxylin and eosin (H&E) for histological evaluation. Epidermal thickness in the dorsal skin and ears was measured in three randomly selected areas of H&E‐stained slides using a microscope (Leica, Hessen, Germany). Mast cell infiltration was assessed by staining dorsal skin sections with toluidine blue, and purple‐stained mast cells were counted in three randomly selected areas.

### 2.13. Immunofluorescence and Immunohistochemistry

Slides were hydrated, and antigen retrieval was performed using sodium citrate. For immunofluorescence, slides were incubated overnight at 4°C with primary antibodies, followed by three PBS washes and a 1‐h incubation with secondary antibodies. Slides were then stained with DAPI (Thermo Fisher Scientific, USA) for 5 min at room temperature, mounted with antifade medium, and coverslipped to preserve fluorescence. For immunohistochemistry, slides were incubated with primary antibodies, followed by incubation with horseradish peroxidase–conjugated rabbit anti‐mouse IgG for 20 min at room temperature.

### 2.14. Quantitative Real‐Time Polymerase Chain Reaction (qRT‐PCR)

Total RNA was extracted from tissue samples using TRIzol reagent (Invitrogen) following the manufacturer’s instructions. RNA concentration and quality were determined using a NanoDrop instrument (Thermo Fisher Scientific). Reverse transcription was performed with 0.5 μg of RNA to synthesize cDNA using a cDNA Synthesis Kit (Thermo Fisher Scientific). qRT‐PCR was conducted using an Applied Biosystems qRT‐PCR system. Reaction mixtures (20 μL) contained 2 μL of cDNA, 10 μL of SYBR Premix Ex Taq II (applied biosystems), 1 μL of each primer (10 μM), and 6 μL of distilled water. The thermal cycling conditions were 95°C for 10 min, followed by 40 cycles of 95°C for 15 s and 60°C for 60 s. Data were normalized to β‐actin and expressed as ratios relative to untreated controls. A list of the verified primers is presented in Table 1.

**Table 1:** qRT‐PCR primer.

Gene name	F/R	Sequence
*h-IL4*	F	CCGTAACAGACATCTTTGCTGCC
	R	GAGTGTCCTTCTCATGGTGGCT

*h-IL13*	F	ACGGTCATTGCTCTCACTTGCC
	R	CTGTCAGGTTGATGCTCCATACC

*h-TSLP*	F	TATCTGGTGCCCAGGCTATTCG
	R	TGAAGCGACGCCACAATCCTTG

*h-IL17*	F	CGGACTGTGATGGTCAACCTGA
	R	GCACTTTGCCTCCCAGATCACA

*h-IL31*	F	ATAGCTCTGCGATGTGCGGTCA
	R	GCTGGTTTCAGGACTCTCCACA

*h-IL22*	F	GTTCCAGCCTTATATGCAGGAGG
	R	GCACATTCCTCTGGATATGCAGG

*h-TNF-α*	F	CTCTTCTGCCTGCTGCACTTTG
	R	ATGGGCTACAGGCTTGTCACTC

*h-IFN-γ*	F	GTCGCCAGCAGCTAAAACAG
	R	CTGGGATGCTCTTCGACCTC

*h-Filaggrin*	F	TGAAGCCTATGACACCACTGA
	R	TCCCCTACGCTTTCTTGTCCT

*h-CCL17*	F	CGGACCCCAACAACAAGAGA
	R	CTCCCTCACTGTGGCTCTTC

*h-CCL22*	F	GAAGCCTGTGCCAACTCTCT
	R	GGGAATCGCTGATGGGAACA

*h-GAPDH*	F	CTGTTGCTGTAGCCAAATTCGT
	R	ACCCACTCCTCCACCTTTGA

*m-IL4*	F	TCACTGACGGCACAGAGCTA
	R	CTTCTCCTGTGACCTCGTT

*m-IL13*	F	TGAGGAGCTGAGCAACATCACACA
	R	TGCGGTTACAGAGGCCATGCAATA

*m-IL22*	F	GCTTGAGGTGTCCAACTTCCAG
	R	ACTCCTCGGAACAGTTTCTCCC

*m-IL31*	F	ACACCGAGTTGGAGAGCCGTAT
	R	CTGTCCTCAGACCGATGTTCTC

*m-CCL17*	F	CGAGAGTGCTGCCTGGATTACT
	R	GGTCTGCACAGATGAGCTTGCC

*m-CCL22*	F	GTGGAAGACAGTATCTGCTGCC
	R	AGGCTTGCGGCAGGATTTTGAG

*m-TSLP*	F	AGCTTGTCTCCTGAAAATCGAG
	R	AGGTTTGATTCAGGCAGATGTT

*m-TNF-α*	F	AACTCCAGGCGGTGCCTATG
	R	TCCAGCTGCTCCTCCACTTG

*m-IFN-γ*	F	AAGCGTCATTGAATCACACC
	R	TGACCTCAAACTTGGCAATA

*m-IL6*	F	ACAAAGCCAGAGTCCTTCAGAG
	R	CATTGGAAATTGGGGTAGGA

*m-IL1-β*	F	CCCTATGGAGATGACGGAGA
	R	GAGCATCTCTTGGATGGCAA

*m-GAPDH*	F	CATTGCTGACAGGATGCAGAAGG
	R	TGCTGGAAGGTGGACAGTGAGG

### 2.15. Western Blot

Proteins were extracted from mouse tissues using radioimmunoprecipitation assay (RIPA) buffer (Thermo Fisher Scientific). Equal amounts of protein (30 μg) were separated on a 10% SDS‐PAGE gel and transferred to nitrocellulose membranes. Membranes were blocked in TBS‐T containing 5% skim milk and incubated overnight at 4°C with primary antibodies. Following five washes with TBS‐T, membranes were incubated with appropriate secondary antibodies for 1 h at room temperature. Protein bands were visualized using a chemiluminescence imaging system (Thermo Fisher Scientific).

### 2.16. Statistics Analysis

Data are expressed as mean ± standard error of the mean (SEM). Statistical significance was determined using one‐way analysis of variance (ANOVA) followed by pairwise comparisons using a modified *t*‐test with Bonferroni correction. Analyses were performed using SPSS, version 12 (SPSS Inc., Chicago, IL, USA), with *p*‐values < 0.05 considered statistically significant.

## 3. Result

### 3.1. iMSC Secretome Isolation and Analysis

The secretome preparation process is illustrated in Figure 1A. After culturing, samples were thoroughly washed with PBS to remove FBS components, confirmed by BSA ELISA (Figure 1B). Post‐washing, the BSA concentration matched that of the PBS washing solution, ensuring complete FBS removal. The secretome weight and total protein content were slightly higher in iMSC‐derived samples compared to MSC‐derived samples (Figure 1C, D). Fluorescence‐activated cell sorting (FACS) analysis showed no changes in cell properties after switching to serum‐free medium, confirming that the medium change did not affect cell characteristics (Figure 1E).

Figure 1Characterization of iPSC derived MSC (iMSC) secretome. (A) The schematic summary of the iMSC and iMSC secretome producton methods. (B) Confirmation of FBS component removal by following PBS method in iMSC culture medium. Detection of FBS components in washed PBS and iMSC secretome using BSA ELISA assay. Proteomic analysis of iMSC secretome and MSC secretome by LC–MS/MS in three independent batches of iMSC secretome. (C) Production weight of iMSC and MSC secretome. (D) Total protein content of iMSC and MSC secretome. (E) Flow cytometry analysis of Passage 4 iMSC and serum‐free media cultured iMSC. The reactivities of iMSC against positive (CD90, CD105, and CD73) or negative (CD34, CD45, and TRA‐1‐60) markers of MSCs were tested. The blue bar labeled as iMSC P4, and the red bar labeled as iMSC secretome P4.

**Figure:**
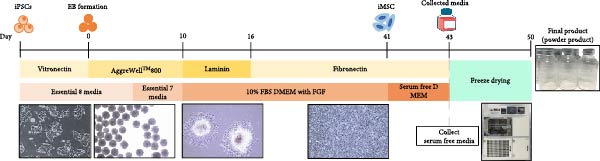
(A)

**Figure:**
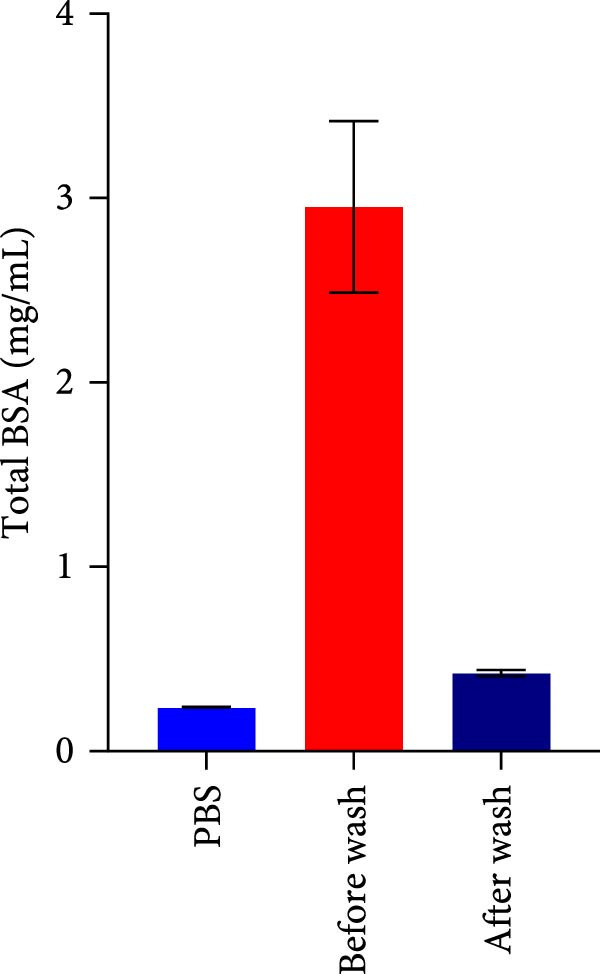
(B)

**Figure:**
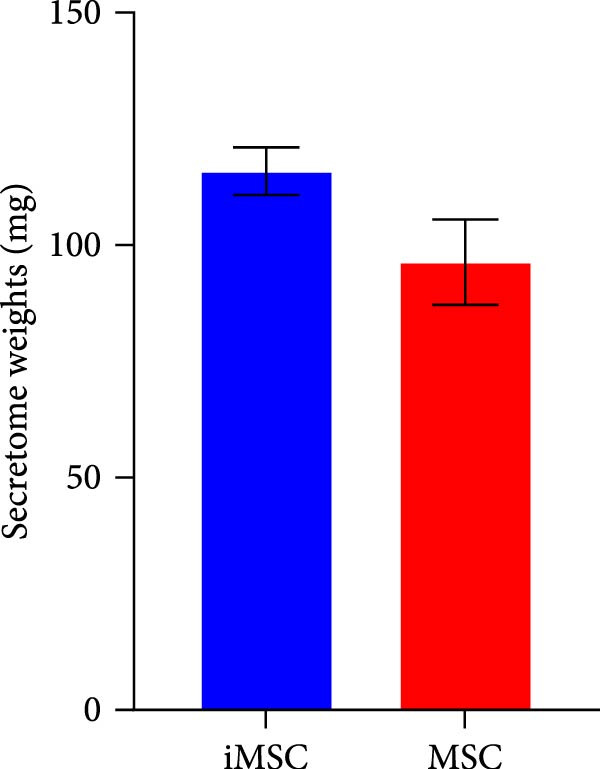
(C)

**Figure:**
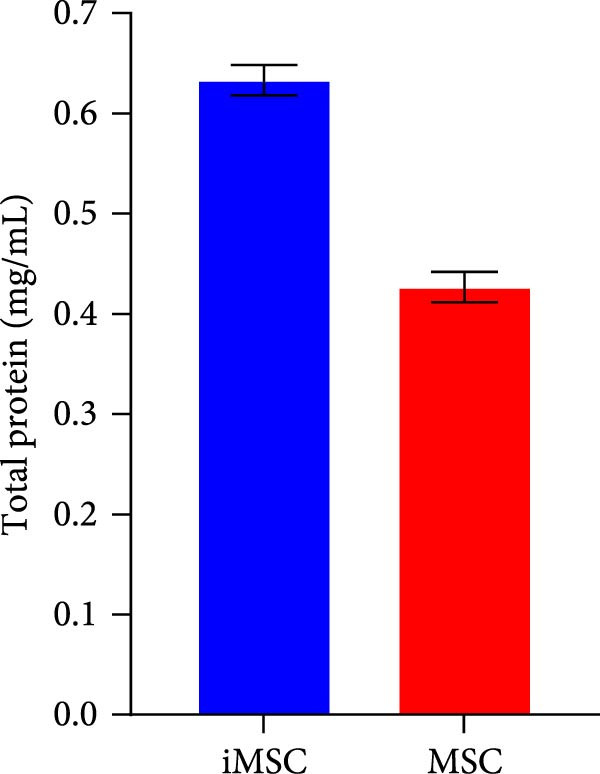
(D)

**Figure:**
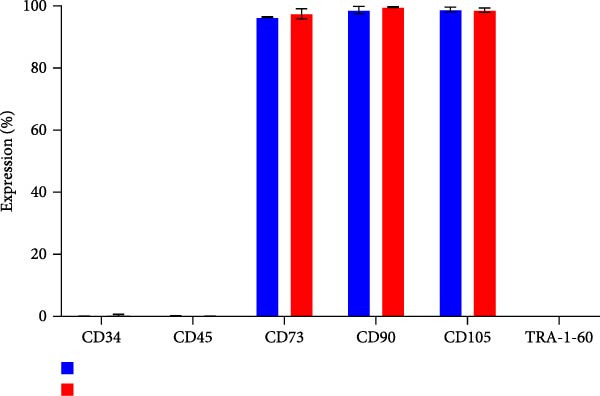
(E)

### 3.2. Profiling of Proteins in iMSC Secretome

Proteomic analysis of three batches of iMSC and MSC secretomes (Figure 2A) using liquid chromatography with tandem mass spectrometry (LC–MS/MS) identified 1474 proteins, with 1022 significantly upregulated in the iMSC secretome. Gene Ontology (GO) analysis indicated enrichment of factors associated with skin regeneration and immune functions, including cell migration, innate immune response, epithelial cell differentiation, wound healing, extracellular matrix organization, and skin development (Figure 2B). Although the iMSC secretome contained a higher total protein content than its MSC counterpart, the observed therapeutic advantages are unlikely to stem from protein quantity alone. Rather, proteomic and pathway analyses point to qualitative differences in secreted bioactive factors as the main contributors to its enhanced efficacy. Notably, 31 of these factors were found to positively influence AD (Table 2). Among them, elastin microfibril interfacer 1 (EMILIN1), transforming growth factor beta (TGF‐β) 1, TGF‐β2, secreted frizzled‐related protein 2 (SFRP2), and integrin beta 1 (ITGB1) were linked to regeneration‐related functions (Figure 2c). Kyoto Encyclopedia of Genes and Genomes (KEGG) pathway analysis further identified the pathways associated with the upregulated factors, including phosphoinositide 3‐kinase/protein kinase B (PI3K/Akt), hypoxia‐inducible factor (HIF) 1, and cyclic adenosine monophosphate (cAMP) signaling and fatty acid metabolism (Figure 2D).

Figure 2Proteomic analysis of iMSC secretome and MSC secretome by LC–MS/MS in three independent batches of iMSC secretome (A) Scatter plot shows the differential expressed gene (DEGs) in iMSC secretome 3 batch average and MSC secretome. Red dots indicate significantly upregulated genes and green dots indicate downregulated genes (B–D). Gene Ontology (GO) analysis of upregulated protein compared to MSC. Enrichment of GO molecular function and cellular component performed using DAVID Bioinformatics resources 6.8. (B) Identification of GO biological pathways of proteins up regulated in iMSC secretome. (C) Results of confirming the correlation of genes shared by GO category. Confirm that genes are shared for each function. (D) Identification of Kyoto Encyclopedia of Genes and Genomes (KEGG) pathways associated with up‐regulated proteins.

**Figure:**
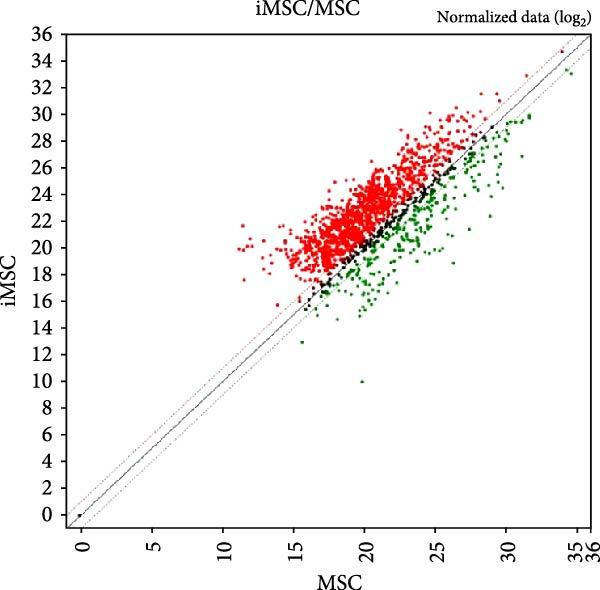
(A)

**Figure:**
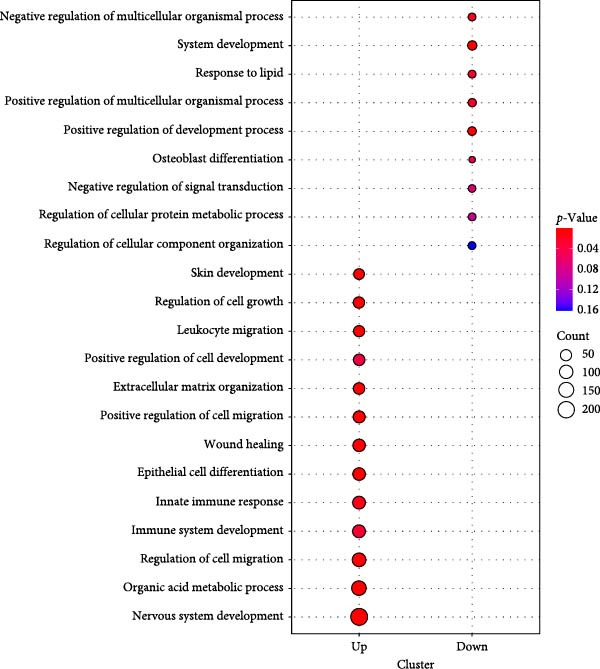
(B)

**Figure:**
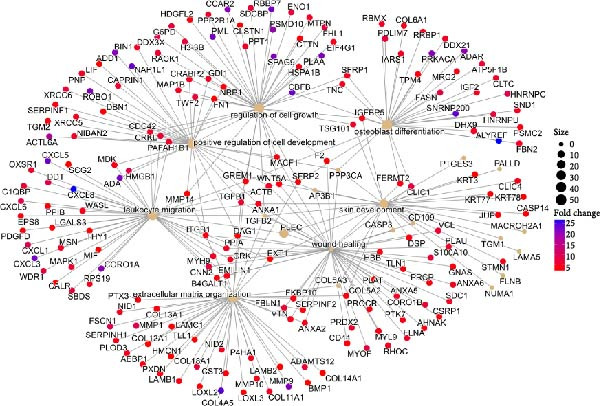
(C)

**Figure:**
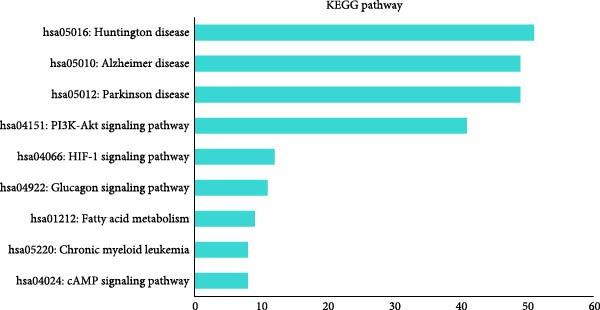
(D)

**Table 2:** Factors that positively affect atopic dermatitis identified through proteomics analysis.

Factor	Function
COL4A5	Atopic dermatitis, rhinitis, asthma, etc. can occur due to Alport syndrome that occurs when a mutation occurs
ACAT1	Attenuates macrophage‐induced inflammation
ECHS1	Related to fatty acid metabolism
FASN	Increased barrier permeability recovery when skin barrier is disrupted
PRDX5	Mitochondrial protein decreased in atopic dermatitis Binds to NRF2 for antioxidant effect and regulates proinflammatory response
PGK1	Anti‐inflammatory action that inhibits the production of “ITI‐H4 isoform,” an inflammatory factor
YWHAG	Induction of oxidative cell death upon reduction, increased oxidative stress, and increased inflammation
SDCBP	Genes related to melanocyte production, miR‐155 expression is increased in skin with vitiligo and SDCBP of melanocytes is decreased (miR‐155: a proinflammatory factor, also increased in skin of atopic patients), involved in keratinocyte cell extension and migration
YWHAZ	Genes mainly secreted by Tregs, increase in anti‐inflammatory cytokines, also effective against arthritis
PPP2R1A	Increased inflammation when mutated
TGF‐β1	Oral supplementation of TGF‐β in children can prevent atopic disease Helps restore keratinocytes, fibroblasts, and vascular connective tissue destruction caused by steroid side effects.
AIMP1	Helps improve wrinkles, whitening, and skin barrier recovery, and increases TH2 immune response in case of deficiency
PKM	Reduces NLRP3 and AIM2 inflammasome responses
LAMA5	Antioxidant action, effective in hair cell proliferation, protein involved in the network that anchors keratin filaments of the cytoskeleton to the dermis at the dermal–epidermal junction, decreased in atopic patients
TGF‐β2	Oral supplementation of TGF‐β in children can prevent atopic disease Helps restore keratinocytes, fibroblasts, and vascular connective tissue destruction caused by steroid side effects
HSP90AA1	HSP90 protein: increased cell proliferation, improved atopic dermatitis, increased KRT10
LAMC2	Helps strengthen the dermal–epidermal junction, and thus, promotes skin firmness
EMILIN1	EMILIN‐1 deficiency promotes chronic inflammatory disease through altered TGF‐β signaling and impaired gC1 q/α4β1 integrin interaction
COL6A1	Mutations can cause skin abnormalities such as dry skin, abnormal scarring, and red streaks
EPRS1	In human inflammatory environments such as pathogenic bacterial infection and inflammatory bowel disease, it activates AKT and promotes IL10 secretion, thereby, alleviating inflammation.
SERPINF2	Inhibitor of KLK, which is highly expressed in atopic patients (KLK7: highly expressed in damaged skin of atopic patients, SERPINF2 is a strong inhibitor of KLK7)
LAMB1	Decreased in atopic patients, members of the laminin family that form an important part of the skin ECM
SFRP2	Restoration of epidermal enhancement
ITGB1	Genes mainly secreted by Tregs, increase in anti‐inflammatory cytokines, also effective against arthritis
LAMC1	Genes related to skin barrier function
FN1	Genes that play a role in wound healing
GNAS	Genes that can cause atopic dermatitis when mutated
PTPRK	STAT3 signaling inhibitor/upregulated in keratinocytes and increases cell proliferation
LAMB2	Genes decreased in atopic patients
LOXL2	Involved in notch signaling expression decreased in atopic dermatitis

### 3.3. Protective Effect of iMSC Secretome Against AD Induction and Wound Healing Assay

The pathogenesis of AD is schematically illustrated in Figure 3A. CCK8 analysis showed that the iMSC secretome significantly protected against apoptosis induced by IL‐4, IL‐13, and IFN‐γ (*p*  < 0.001), key cytokines in AD pathogenesis (Figure 3B). Pretreatment with iMSC secretome before AD induction reduced mRNA expression levels of TNF‐α, IFN‐γ, IL‐4, IL‐13, IL‐17, IL‐22, IL‐31, CCL17, CCL22, and TSLP, while increasing filaggrin mRNA levels (*p*  < 0.0001), compared to MSC secretome treatment or untreated controls (Figure 3C).

Figure 3iMSC secretome exhibits atopic dermatitis protective effects in HaCaT cells stimulated with IL4, TNF‐α, and IFN‐γ. (A) The developmental stage of a skin disease known as atopic dermatitis. (B–E) The protective effect of iMSC secretome on atopic dermatitis was confirmed 24 h after pretreatment with iMSC secretome (50 and 200 μg/mL), tacrolimus (1 μg/mL), and MSC (200 μg/mL) for 2 h, followed by treatment with IL4 (30 ng/mL) and TNF‐α/IFN‐γ (10 ng/mL each). (B) Results of confirming cell viability through CCK assay. (C) The relative expression levels of the atopic dermatitis associated genes were evaluated by qRT‐PCR: IL4, IL13, IL17, IL22, IL31, CCL17, CCL22, TSLP, filaggrin, TNF‐α, and IFN‐γ. (D, E) The protective effect of iMSC secretome against atopic dermatitis was confirmed through the wound healing assay. Data are presented as mean ± SEM.  ^∗^ *p*  < 0.05;  ^∗∗^ *p*  < 0.01;  ^∗∗∗^ *p*  < 0.001,  ^∗∗∗∗^ *p*  < 0.0001.

**Figure:**
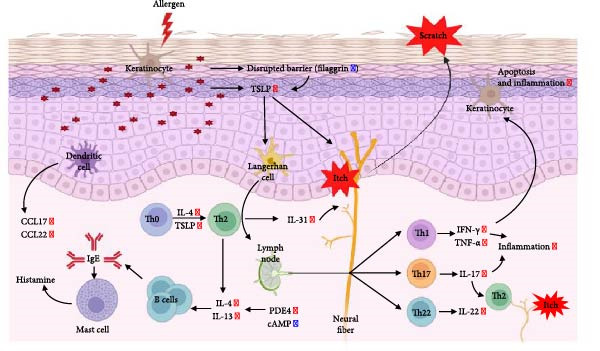
(A)

**Figure:**
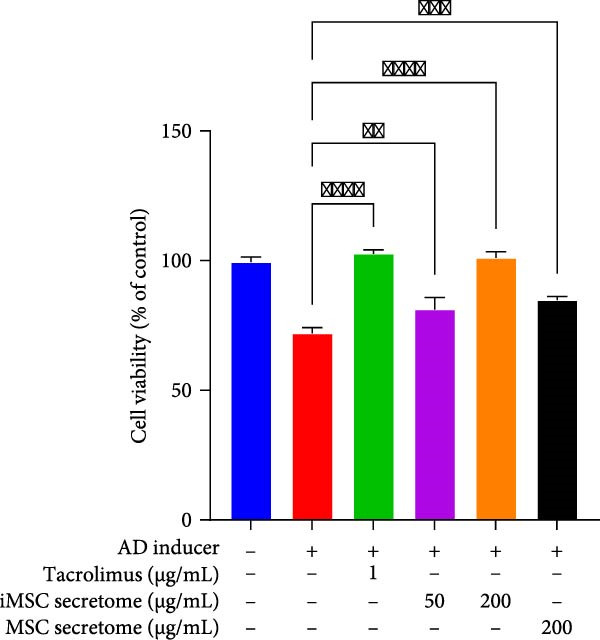
(B)

**Figure:**
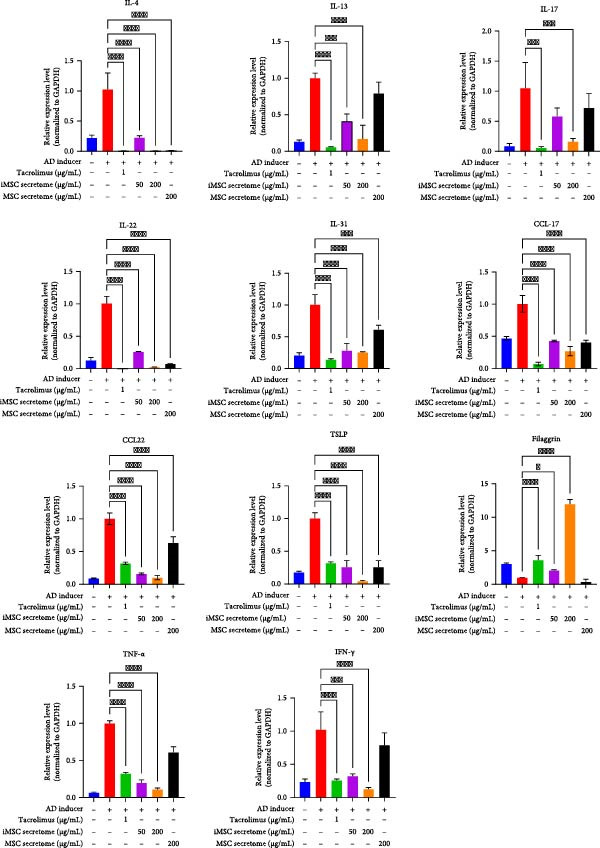
(C)

**Figure:**
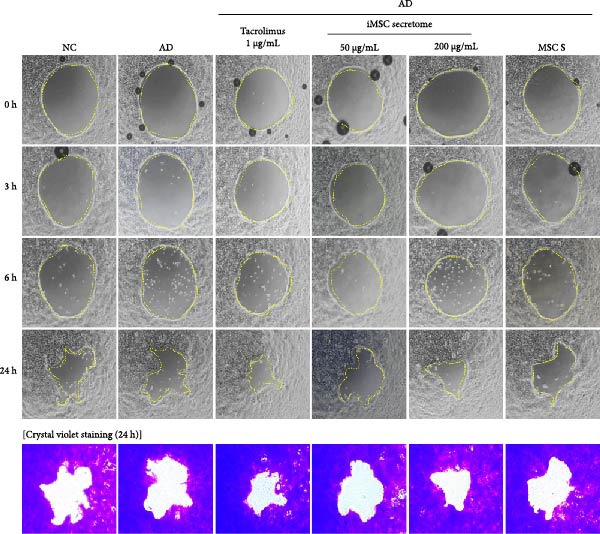
(D)

**Figure:**
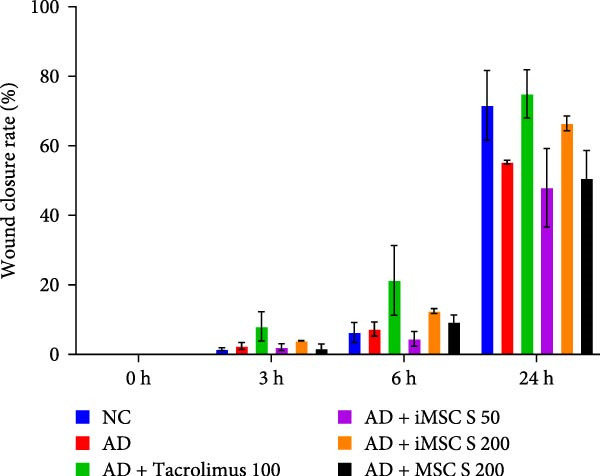
(E)

In a wound healing assay, scratched HaCaT cells pretreated with 200 μg/mL iMSC secretome exhibited a higher cell migration rate, reflecting improved wound healing, compared to the AD and MSC secretome groups (Figure 3D, E).

### 3.4. iMSC Secretome Clinically Alleviates AD

The therapeutic potential of the iMSC secretome was evaluated using a DNCB‐induced AD mouse model in NC/Nga mice, as outlined in the schematic in Figure 4A. The model successfully replicated AD symptoms, including erythema, edema, scaling, and excoriation (Figure 4B).

Figure 4iMSC secretome improve atopic dermatitis (AD) induced by repeated exposure to DNCB. (A) Schematic diagram of the study protocol. The first 2 days 1% DNCB was applied for sensitization. Tacrolimus (0.1% protopic) was topically applied thrice a week as a positive control. (B) Representative dorsal skin photographs of each treatment group showing comparison of AD‐like skin lesions. (C) The results of checking the severity of skin condition periodically. (D) The results of checking the thickness of the ears periodically. (E) Dorsal skin sections were stained with hematoxylin–eosin (H&E) and toluidine blue. The epidermis thickness was measured in H&E slides and the mast cells were stained purple (black arrows) in toluidine blue‐stained (TB) slides. (F) The results of measuring the thickness of the epidermis. (G) The result of checking the number of mast cells. (H) Total levels of serum IgE, IL4, IL13, IL31, IL6, and IL1‐β were measured by ELISA. *n* = 6. Data are presented as mean ± SEM.  ^∗^ *p*  < 0.05;  ^∗∗^ *p*  < 0.01;  ^∗∗∗^ *p*  < 0.001,  ^∗∗∗∗^ *p*  < 0.0001.

**Figure:**
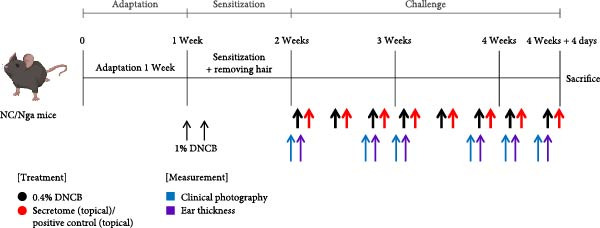
(A)

**Figure:**
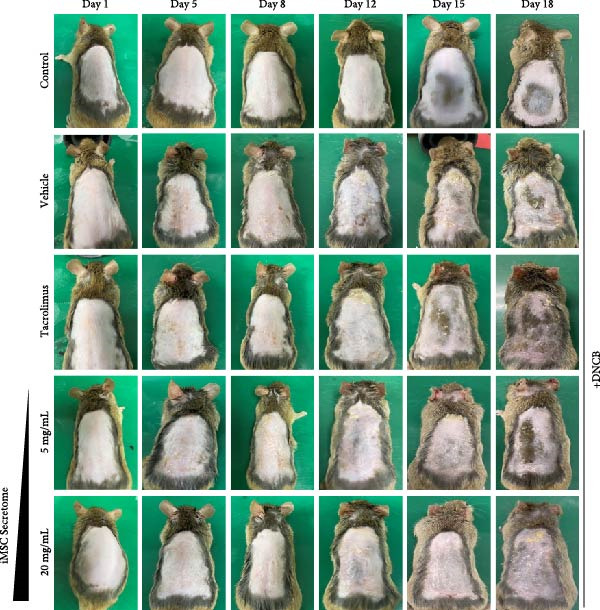
(B)

**Figure:**
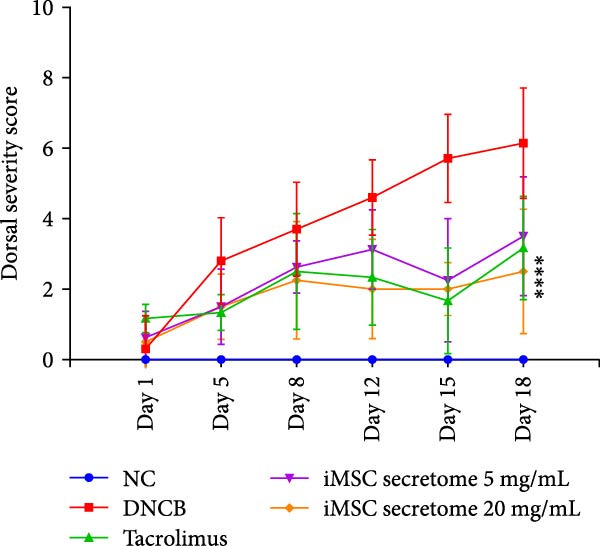
(C)

**Figure:**
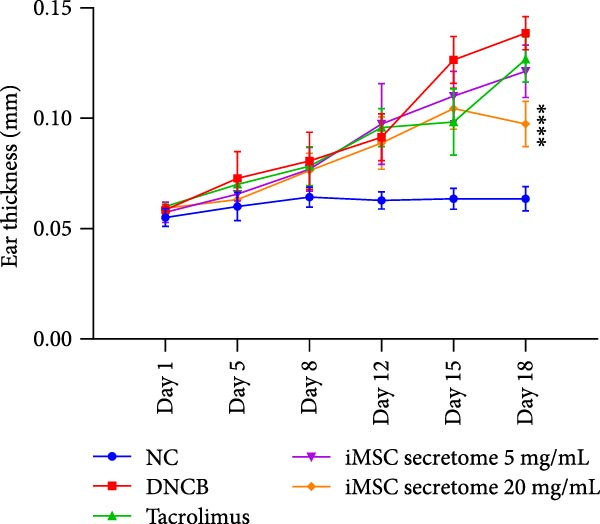
(D)

**Figure:**
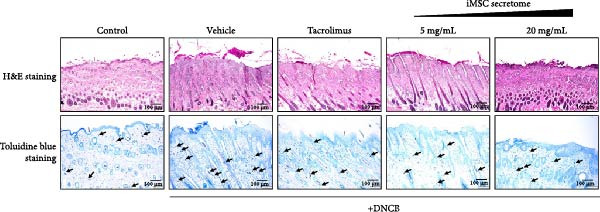
(E)

**Figure:**
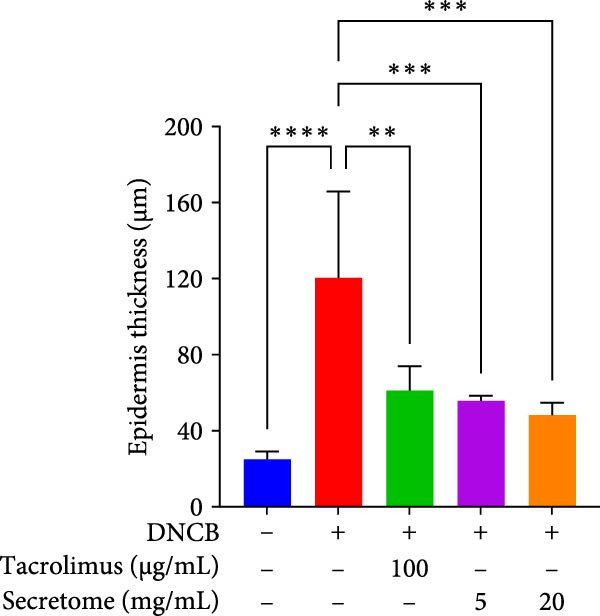
(F)

**Figure:**
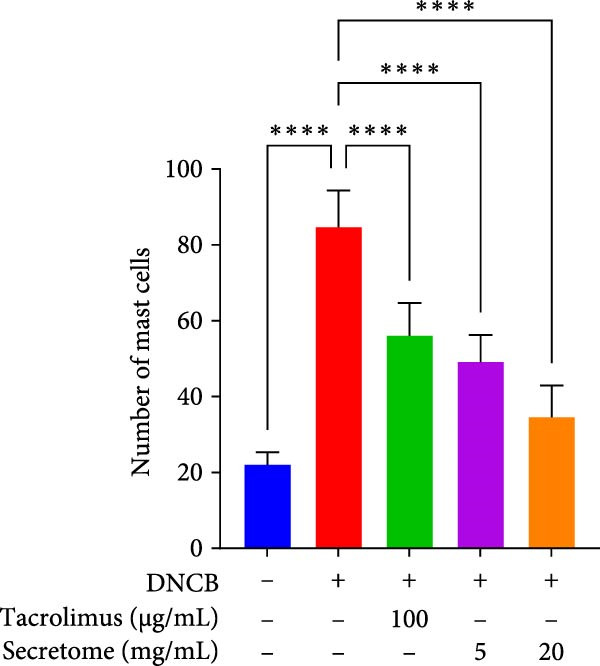
(G)

**Figure:**
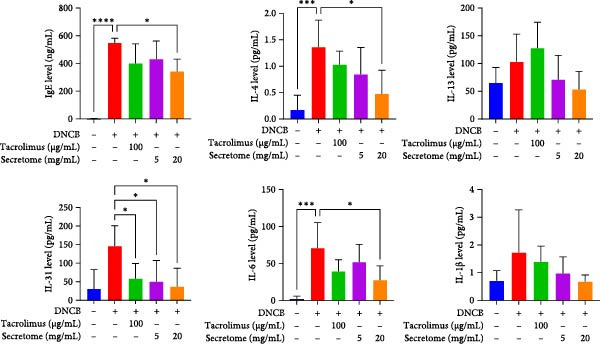
(H)

DNCB exposure during the challenge period significantly increased dorsal skin severity scores and ear thickness (*p*  < 0.0001), both of which were markedly reduced with iMSC secretome treatment (Figure 4C, D). Histological analysis revealed increased epidermal thickness in the DNCB group, which significantly decreased following iMSC secretome treatment, consistent with the reduced skin severity scores (*p*  < 0.001) (Figure 4E, F). Additionally, mast cell counts in the dermis were significantly higher in the DNCB group compared to controls and were significantly reduced with iMSC secretome treatment (*p*  < 0.0001) (Figure 4E, G).

### 3.5. iMSC Secretome Reduces Inflammation and Restores Skin Barrier in AD Model Mice

In the AD model mice, IgE production and Th2‐related cytokines (IL‐4 and IL‐31), along with the inflammatory cytokine IL‐6, were significantly elevated, effectively replicating AD pathology (*p*  < 0.05). These inflammatory responses were markedly reduced with iMSC secretome treatment (Figure 4H).

qRT‐PCR analysis revealed significant decreases in AD biomarkers (IL‐4, IL‐13, IL‐22, IL‐31, CCL17, and TSLP) and inflammatory cytokines (TNF‐α, IFN‐γ, IL‐6, and IL‐1β) in the iMSC secretome treatment groups (5 and 20 mg/mL) compared to the DNCB group (*p*  < 0.05) (Figure 5A). IHC staining confirmed that the elevated expression of Th2‐related cytokines (IL‐4, IL‐13, and IL‐31) induced by DNCB was significantly reduced following iMSC secretome treatment (*p*  < 0.05) (Figure 5B–E).

Figure 5iMSC secretome ameliorates histological changes in DNCB‐induced atopic dermatitis lesions. (A) Effects of iMSC secretome on the mRNA expression levels of atopic dermatitis biomarkers and inflammatory cytokines in DNCB‐induced skin lesions. The relative mRNA expression levels of Il4, IL13, IL22, IL31, CCL17, CCL22, and TSLP. (B) Immunohistochemical (IHC) staining of mouse skins using antibodies against mouse IL4, IL13, and IL31. (C–E) Representative graph. (F) Filaggrin expression analysis as determined by immunofluorescence and western blot in DNCB induced AD mice model. (G) Evaluation of the efficacy of improving side effects of tacrolimus as a positive control, western blot analysis of CGRP, and substance P expression in skin tissues of AD mice that received iMSC secretome. *n* = 6. Data are presented as mean ± SEM.  ^∗^ *p*  < 0.05;  ^∗∗^ *p*  < 0.01;  ^∗∗∗^ *p*  < 0.001,  ^∗∗∗∗^ *p*  < 0.0001.

**Figure:**
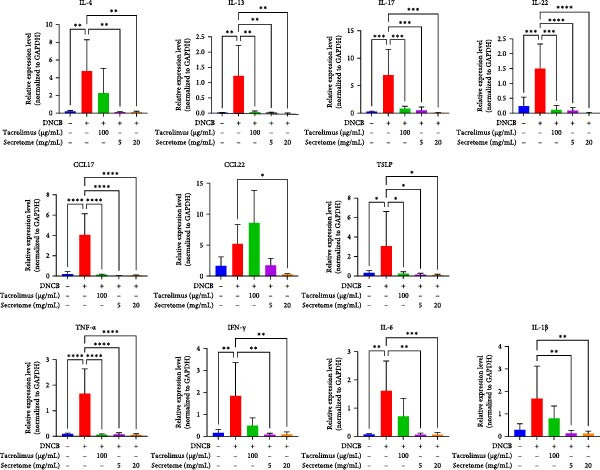
(A)

**Figure:**
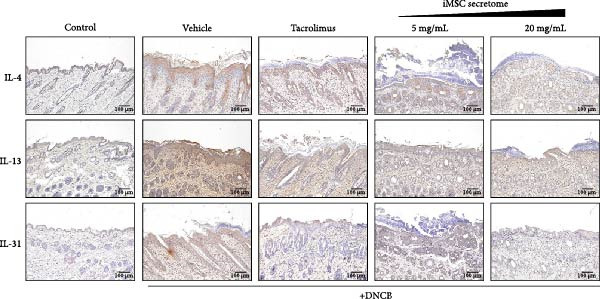
(B)

**Figure:**
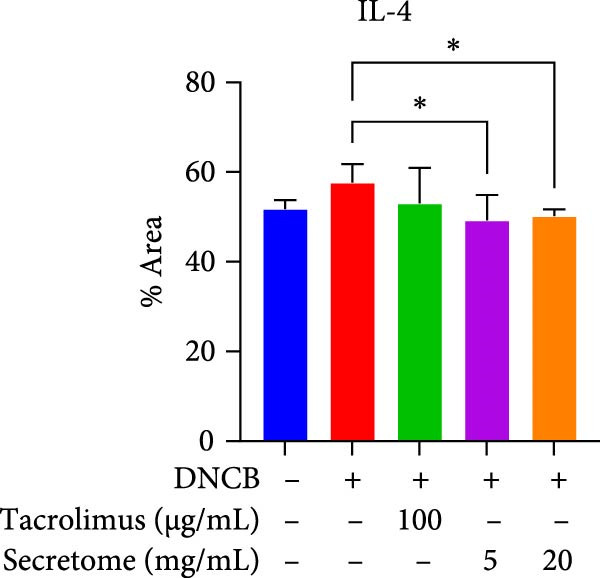
(C)

**Figure:**
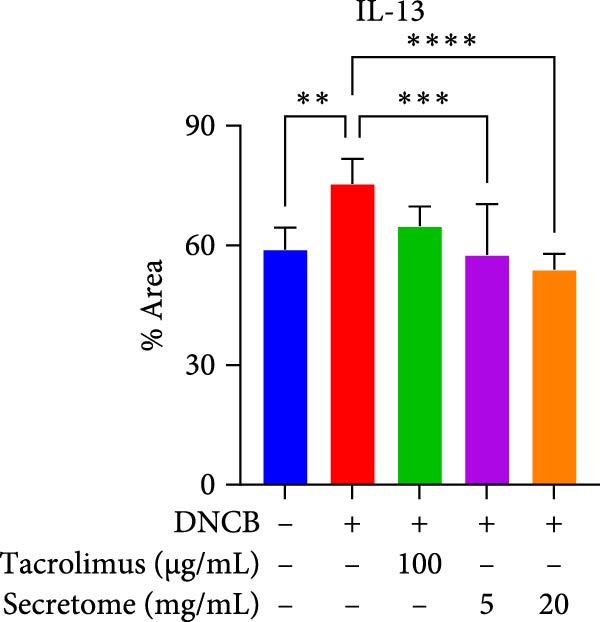
(D)

**Figure:**
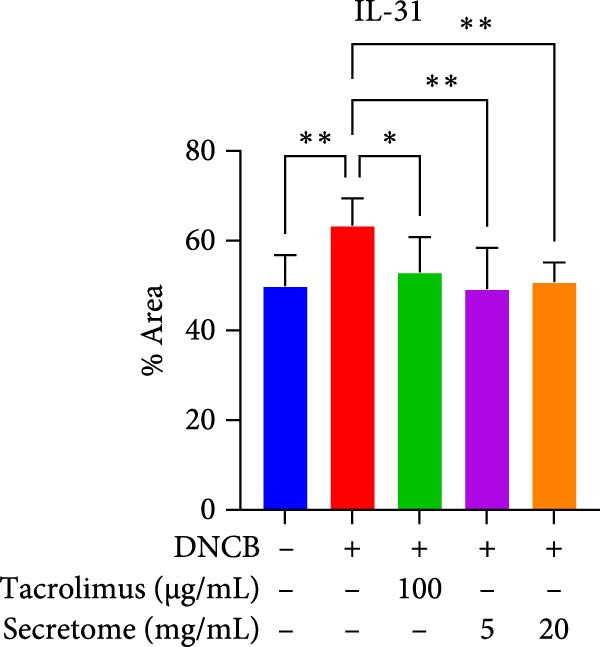
(E)

**Figure:**
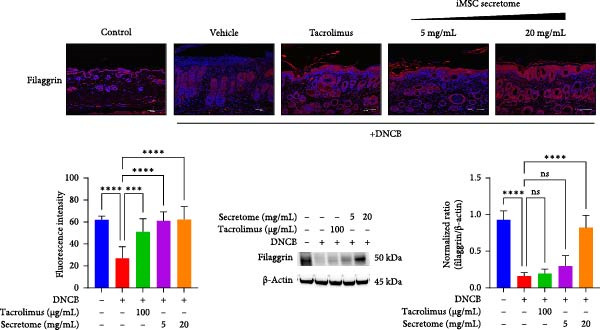
(F)

**Figure:**
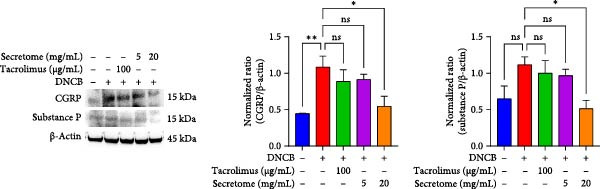
(G)

To evaluate skin barrier restoration, immunofluorescence, and western blot analyses were conducted to assess filaggrin expression. Both analyses showed a significant increase in filaggrin expression, which was suppressed by DNCB treatment, after treatment with 20 mg/mL iMSC secretome (*p*  < 0.0001) (Figure 5F). Tacrolimus and the 5 mg/mL iMSC secretome were found to enhance filaggrin expression in immunofluorescence images, and a similar trend was observed in the corresponding western blot bands. However, some differences in the degree of expression were noted, which may be attributed to variations in detection sensitivity, protein localization, or technical factors such as sample loading or antibody affinity.

### 3.6. iMSC Secretome Reduces Neuropeptides

Western blot analysis revealed significant reductions in the neuropeptides calcitonin gene‐related peptide (CGRP) and substance P following treatment with 20 mg/mL iMSC secretome (*p*  < 0.05) (Figure 5G). These neuropeptides play key roles in AD‐related inflammation, distress, vasodilation, plasma extravasation, itching, and burning sensations, which are also linked to TCI.

### 3.7. iMSC Secretome Recovered Altered Gene Expression

To evaluate the impact of iMSC secretome on skin gene expression during AD treatment, proteomics analysis was performed on skin lesions from the control, vehicle, Tacrolimus, and iMSC secretome groups. Principal component analysis (PCA) showed clear separation of the iMSC secretome‐treated groups from the other groups, indicating activation of a distinct gene expression program during AD recovery (Figure 6A).

Figure 6iMSC secretome modulates distinct gene expression program during AD pathogenesis. (A) The principal component analysis (PCA) showing the first two principal components of RNA‐Seq data regarding their correlation. (B) Heatmap showing 1802 differential expressed gene (DEGs). (C) Bubble plot shows the significantly (*p*  < 0.05) altered GO biological processes in vehicle, tacrolimus, and iMSC secretome treated groups compared with the vehicle group. The size of the bubble is proportional to the number of genes. Red bubbles indicate upregulation and blue bubbles denote downregulation. GO analysis was performed using DAVID bioinformatics resources 6.8 (https://david.ncifcrf.gov). (D) Heat map of DEGs between vehicle and iMSC secretome that mapped to the indicated GO in skin barrier, lipid metabolism, immune response, and apoptosis.

**Figure:**
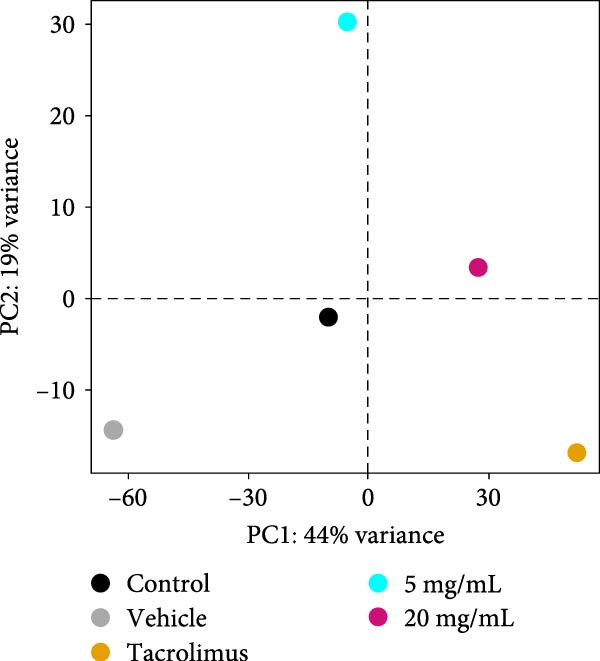
(A)

**Figure:**
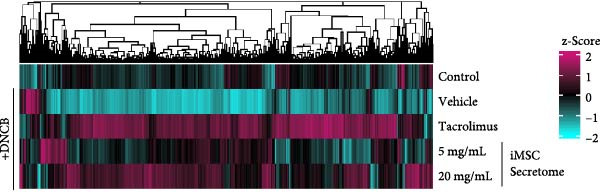
(B)

**Figure:**
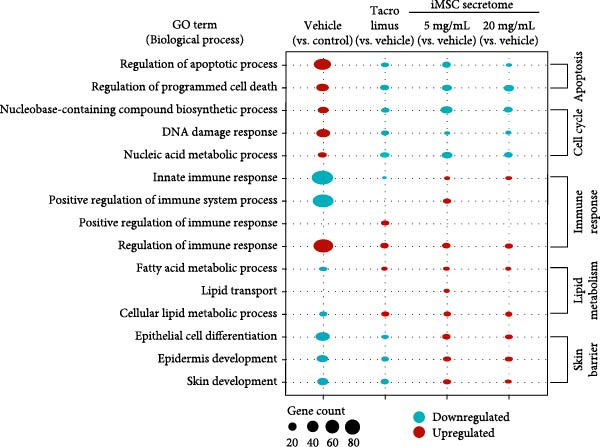
(C)

**Figure:**
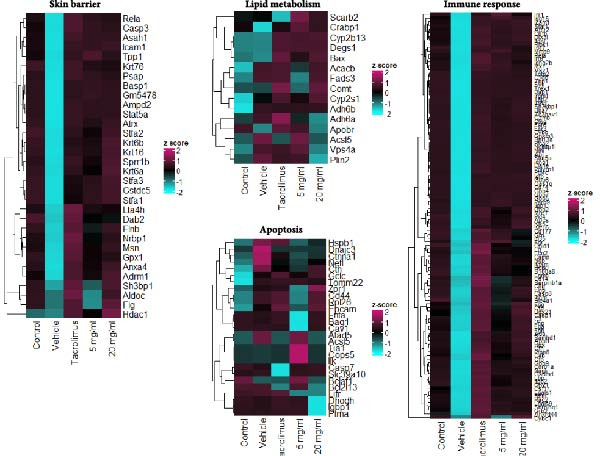
(D)

Analysis identified 2829 differentially expressed genes (DEGs) in the epidermis across groups, with a fold change >2 (Figure 6B). GO analysis revealed that iMSC secretome treatment restored genes related to skin barrier functions, including skin development, epidermis development, and epithelial cell differentiation, which were downregulated in the vehicle group (Figure 6C, D). Additionally, lipid metabolic processes, such as cellular lipid metabolism, lipid transport, and fatty acid metabolism, were significantly activated.

The iMSC secretome also downregulated genes associated with transporters involved in cell cycle regulation and apoptosis responses. In contrast, Tacrolimus treatment partially reversed DNCB‐induced gene expression changes and demonstrated limited effects on restoring skin barrier function.

## 4. Discussion

This study highlights the promise of the iMSC secretome in treating AD. First, proteomic and GO analyses provided molecular evidence, revealing its roles in immune regulation and skin barrier restoration, consistent with KEGG pathway findings. Second, in vitro studies confirmed that the iMSC secretome prevents keratinocytes from releasing AD‐related cytokines upon exposure to AD inducers. Third, in vivo efficacy in reducing inflammation and enhancing skin barrier was demonstrated by clinical, histological, and immunological improvement in the AD mouse model.

This study is significant as it not only investigated the efficacy of the iMSC secretome in AD, but also confirmed its superiority over the MSC secretome in several aspects. MSCs have been shown to significantly improve AD through their immunomodulatory actions on T and B cell activation, mast cell degranulation, and IgE production [22]. Recent studies have suggested that MSC secretome is responsible for MSC’s therapeutic effects, potentially offering even enhanced efficacy while addressing the limitations of using MSCs themselves [14, 23–27]. However, MSC secretome faces challenges, including the limited availability of homogeneous MSCs, aging of primary MSCs during prolonged culture, reduced yield of secretome during transfer, and difficulties in large‐scale production [28, 29]. The iMSC secretome exhibits the therapeutic effects of MSCs and their secretome while overcoming many of their limitations [30]. Several studies have reported its consistency, reduced heterogeneity, ability to rejuvenate without cellular aging throughout the culture process, and potential to restore skin barrier integrity [31–33]. According to our results, CCK8 analysis revealed iMSC secretome’s superior ability to protect HaCaT cells from AD‐induced apoptosis. Additionally, when challenged with AD inducers, iMSC secretome pretreatment significantly reduced the mRNA levels of cytokines involved in AD pathogenesis (IL‐4, IL‐13, IL‐17, IL‐22, IL‐31, CCL17, CCL22, TSLP, TNF‐α, and IFN‐γ) and increased filaggrin expression in HaCaT cells, outperforming the MSC secretome.

The pathogenesis of AD involves a complex interplay between various cells, including keratinocytes, fibroblasts, and endothelial cells, which are activated by inflammatory stimuli. Central to this inflammatory process is the recruitment of Th2 cells, which produce the key drivers of inflammation in AD, such as IL‐4, IL‐13, and IL‐31 [4]. These cytokines promote the production of serum IgE and eosinophil infiltration into the skin, contributing to AD progression and exacerbation. Additionally, proinflammatory and Th1 cytokines such as IFN‐γ, IL‐6, and IL‐1β, are upregulated, further amplifying inflammation and chronicizing AD. This cascade of immune responses leads to chronic inflammation, skin barrier dysfunction, and pruritus, hallmark features of AD [34, 35]. Our results demonstrated that the iMSC secretome alleviates the key immune reactions of AD, including the expression of Th2 cytokines (L‐4, IL‐13, IL‐31, IL‐22, CCL17, CCL22, and TSLP), as well as other inflammatory cytokines (TNF‐α, IL‐1β, IL‐6, and IFN‐γ). Moreover, the iMSC secretome was shown to promote cell migration in the wound healing assay and enhance filaggrin expression both in cultured keratinocytes and in lesional skin tissues, thereby, contributing to skin barrier restoration. These findings highlight the dual effects of iMSC secretomes in AD, immune modulation and skin barrier regeneration.

These dual effects are further supported by the results of proteomic, GO, and KEGG analyses. KEGG provides a systematic framework for analyzing gene functions within biological systems [36]. Relevant pathways implicated in AD include PI3K/Akt, HIF‐1, cAMP signaling, and fatty acid metabolism. The PI3K/Akt pathway mediates skin development, homeostasis, and regulates cell proliferation, migration, and differentiation, with potential as a therapeutic target for inflammatory skin disorders such as AD, psoriasis, and alopecia [37]. Elevated intracellular cAMP by phosphodiesterase 4 inhibition reduces inflammatory molecule production in various cell types, including T cells and keratinocytes [38]. HIFs regulate filaggrin expression, epidermal barrier function, and innate immunity, while epidermal HIF‐1α deficiency disrupts the barrier and increases allergen sensitivity [39, 40]. Fatty acid metabolism is critical for maintaining epidermal permeability; its disruption exacerbates barrier dysfunction, water loss, and inflammation [41]. Collectively, the iMSC secretome enhances skin homeostasis, reduces inflammation, and strengthens the epidermal barrier through these pathways. In addition, key factors identified within the corresponding KEGG pathways include ITGB1, HSP90AB1, THBS1, and LAMA5, involved in the PI3K‐Akt, cAMP, and HIF‐1 pathways. These factors are associated with positive regulation of cell migration, development, and wound healing in GO pathways. Also, FASN, a factor involved in fatty acid metabolism, supports epithelial cell differentiation within the GO pathways.

The iMSC secretome was also found to reduce the number of mast cells and the level of the neuropeptides (substance P and CGRP) in lesional skin. Mast cells are activated by allergens and IgE/FcεRI cross‐linking, triggering the subsequent allergic reactions in AD [42]. Mast cell degranulation has been reported to be associated with the severity of AD and is one of the mechanisms by which MSCs improve AD [22, 43]. A recent study also reported its role in initiating type 2 inflammation [44]. The ability to reduce the number of mast cells suggests that the iMSC secretome may suppress allergic reactions and type 2 inflammation, alleviating AD at a higher‐level than inhibiting degranulation. Substance P and CGRP are key neuropeptides involved in promoting inflammation and itching in AD. Upon release from nerve endings, they contribute to vasodilation, plasma leakage, chemotaxis of inflammatory cells, and pruritus, while also mediating the local adverse effects of TCI [45, 46]. Approximately 50% of TCI‐treated patients experience a burning sensation, a side effect attributed to the neuropeptide release and mast cell degranulation [46]. In our in vivo experiments, the iMSC secretome more effectively reduced the expression levels of key AD biomarkers (IL‐4, IL‐13, IL‐31, IL‐22, CCL17, CCL22, and TSLP) and other inflammatory cytokines (IFN‐γ, TNF‐α, IL‐1β, and IL‐6) in lesional skin compared to 0.03% Tacrolimus. These findings indicate that the iMSC secretome may effectively mitigate the neuropeptide‐driven inflammation and pruritus in AD, avoiding the side effects associated with TCI.

This study has several limitations, including its preclinical nature and potential variability in the composition of secretome batches, which may affect the consistency of therapeutic outcomes. Future research should focus on translating these findings into clinical settings through well‐designed clinical trials. In parallel, further optimization of the iMSC secretome could enhance its clinical applicability. Approaches such as refining culture conditions, selecting defined induction protocols, and enriching specific bioactive components, including exosomes, cytokines, or growth factors. Additionally, the identification and isolation of the most active molecular components could facilitate the development of more targeted, scalable, and safe therapeutic formulations.

## Disclosure

This study was reviewed and approved by the IACUC in the School of Medicine, The Catholic University of Korea (Approval #CUMC‐2023‐0242‐07).

## Conflicts of Interest

The authors declare no conflicts of interest.

## Author Contributions

Seungah Yoo and Hyun Jin Baek contributed equally to this work.

## Funding

This research was supported by a grant from the Korea Health Technology R&D Project through the Korea Health Industry Development Institute (KHIDI), funded by the Ministry of Health and Welfare, Republic of Korea (Grant HI22C1314); by the Korean Fund for Regenerative Medicine funded by Ministry of Science and ICT, and Ministry of Health and Welfare (Grant RS‐2022‐00165160, Republic of Korea); by the Korea Technology and Information Promotion Agency for SMEs (TIPA), Ministry of SMEs and Startups, Tech Investor Program for Scale‐up (TIPS) (Project Number RS‐2023‐00302955); by the National Research Foundation of Korea (NRF) from the Korean Government (Grant NRF‐2021R1C1C2004688); by a grant of the Manufacturing Human Cells‐based Artificial Blood and Platform Technology Department for Transfusion, funded by Multi‐Ministrial Research Project, Republic of Korea (Grant RS‐2023‐KH142779); by the Basic Medical Science Facilitation Program through the Catholic Medical Center of the Catholic University of Korea funded by the Catholic Education Foundation; and by a grant of the Korea Health Technology R&D Project through the Korea Health Industry Development Institute (KHIDI), funded by the Ministry of Health & Welfare, Republic of Korea (Grant RS‐2025‐16068032).

## Data Availability

The data that support the findings of this study are available from the corresponding author upon reasonable request.
